# Verrucous Carcinoma on the Lateral Border of the Tongue: A Case Report

**DOI:** 10.7759/cureus.96030

**Published:** 2025-11-03

**Authors:** Junu Ojha, Neiha Dhanoa, Weaam Hazime, Clara Poparad-Stezar

**Affiliations:** 1 Division of Integrated Biomedical Sciences, School of Dentistry, University of Detroit Mercy, Detroit, USA; 2 General Dentistry, School of Dentistry, University of Detroit Mercy, Detroit, USA; 3 General Dentistry (Private Practice), School of Dentistry, University of Detroit Mercy, Detroit, USA; 4 Oral and Maxillofacial Surgery, Henry Ford Hospital, Detroit, USA

**Keywords:** oral cancer, papillary, squamous cell carcinoma, squamous papilloma, verrucous carcinoma

## Abstract

Verrucous carcinoma (VC) is a low-grade variant of squamous cell carcinoma (SCC). It presents as a cauliflower-like growth with warty surface projections. VC can appear both extraorally and intraorally. We report a case of a 73-year-old female who presented with an exophytic, verrucopapillary lesion on the left lateral border of her tongue. Her symptoms included difficulty with speech and discomfort while eating. Clinical and histopathological findings rendered a diagnosis of oral verrucous carcinoma (OVC). OVC shares a clinical resemblance to several other papillary lesions. Thus, timely and accurate diagnosis is essential as OVC carries the potential for transformation into SCC.

## Introduction

Malignancies of the oral cavity and pharynx account for approximately 3% of all cancers diagnosed every year in the United States. In 2025, oral cancer was estimated to cause death in 12,770 individuals [1]. Over 90% of oral cancers originate in the squamous cells of epithelial tissue and are classified as squamous cell carcinomas (SCCs) [2]. In 1948, Ackerman described verrucous carcinoma (VC) as a well-differentiated, low-grade variant of SCC. In contrast to the other variants of SCC, oral verrucous carcinoma (OVC) tends to locally invade bone, muscle, cartilage, and salivary glands rather than metastasize to distant areas [3, 4].

The purpose of this article is to present a case of OVC on the lateral border of the tongue and provide insight into the clinical differential diagnoses of OVC. Many other papillary lesions very closely resemble OVC clinically, thereby delaying the diagnosis of OVC. The clinical differential diagnoses of OVC include benign entities such as squamous papilloma, condyloma acuminatum, verruciform xanthoma, and verruca vulgaris. OVC is also known as *Snuff Dipper’s Cancer*, due to its strong association with smokeless tobacco use. Additionally, it often develops in patients with a history of tobacco smoking, proliferative verrucous leukoplakia (PVL), and human papillomavirus (HPV) [2,3]. While OVC can manifest anywhere in the oral cavity, lesions are often localized to the site of chronic tobacco placement [2]. Ample sampling and timely diagnosis of the lesion are crucial because 20% of OVC lesions may have foci of conventional SCC embedded within the lesion [4]. In addition, the treatment and prognosis of SCC, in contrast to OVC, are more extensive and guarded.

## Case presentation

A 73-year-old Asian female presented with a chief complaint of “a swelling and discomfort on the side of my tongue.” The patient first noticed the lesion six months earlier, with an increase in size within the last two months. The increased size of the lesion led to the patient developing symptoms of speech alteration and discomfort while eating. There was no complaint of spontaneous pain or bleeding in the area. The patient’s medical history included hypertension and hyperlipidemia for which she was taking Atenolol 50 mg and Simvastatin 20 mg, respectively. She had denied any history of smoking or alcohol consumption. Her dental history included missing and decayed teeth due to caries and advanced periodontitis.

**Figure 1:**
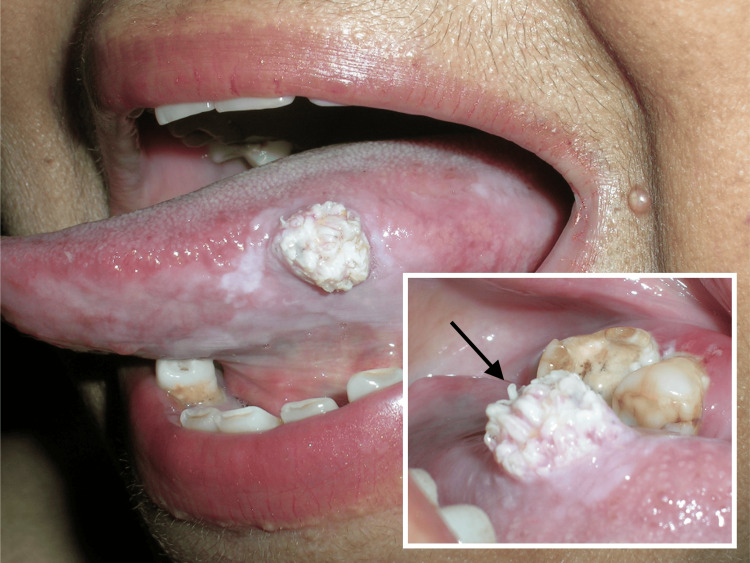
Intra-oral photographs of the lesion. A white, cauliflower-shaped lesion on the left lateral border of the tongue.

Extraoral findings were unremarkable, with no evidence of asymmetry, cellulitis, or lymphadenopathy. Intraoral examination revealed a 2 x 2 x 2 cm sessile, thickened, white, raised, cauliflower-shaped, papillary lesion on the left lateral border of the tongue (Figure 1). There was no evidence of any source of trauma that could have caused this lesion. Additionally, flat white lesions were noted peripheral to the raised white area. Based on the clinical presentation of the lesion, the differential diagnoses included squamous papilloma, condyloma acuminatum, verruciform xanthoma, and verruca vulgaris. After obtaining informed consent addressing the risk assessment, additional testing, research use, and treatment options, an excisional biopsy was performed and submitted for histopathologic evaluation.

**Figure 2:**
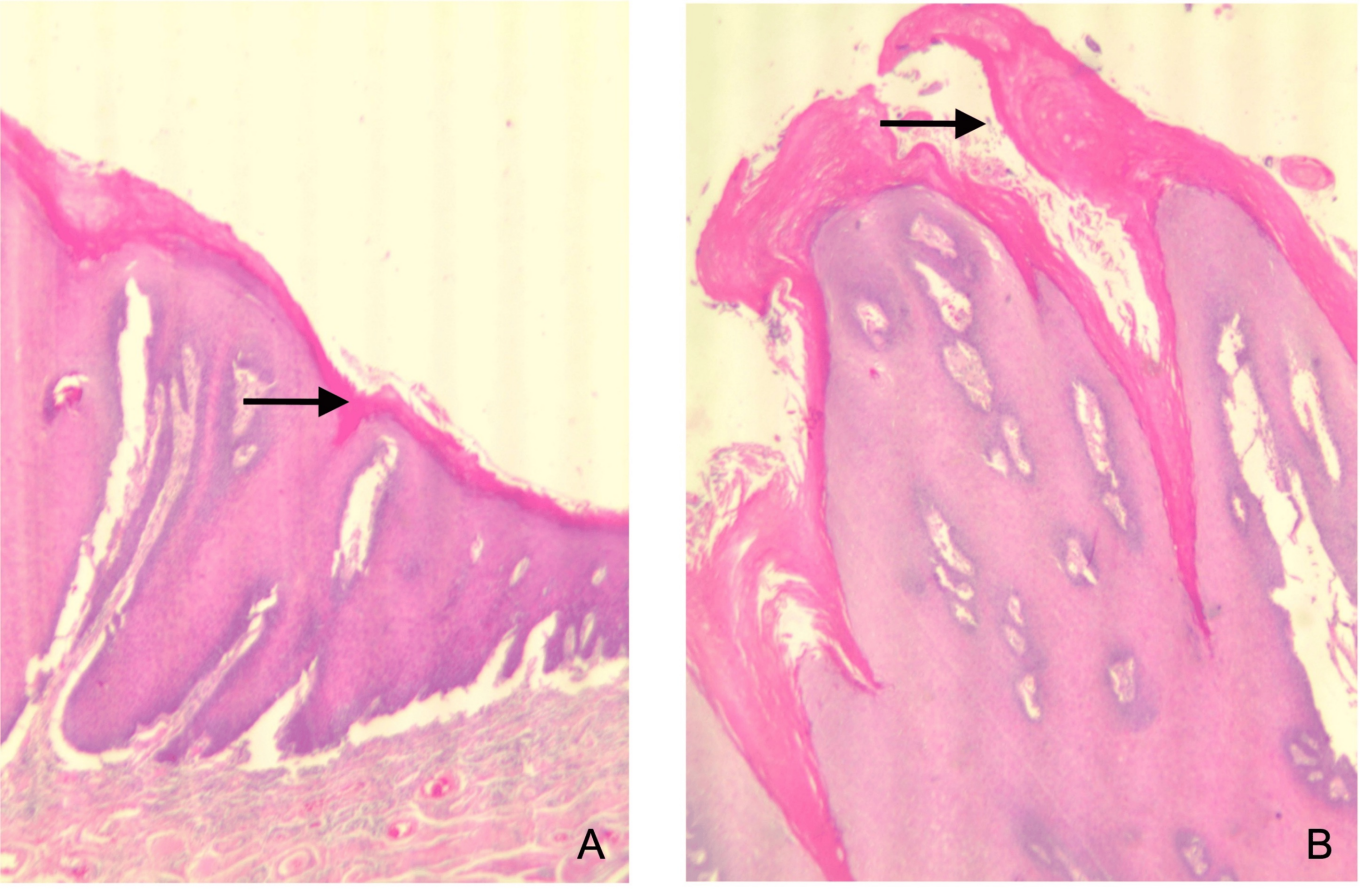
Photomicrographs: (A) stratified squamous epithelium exhibiting a severely thickened parakeratin layer with papillary architecture (H&E, magnification 10x); (B) severely thickened parakeratin layer exhibiting extensive papillary architecture (H&E, magnification 20x).

**Figure 3:**
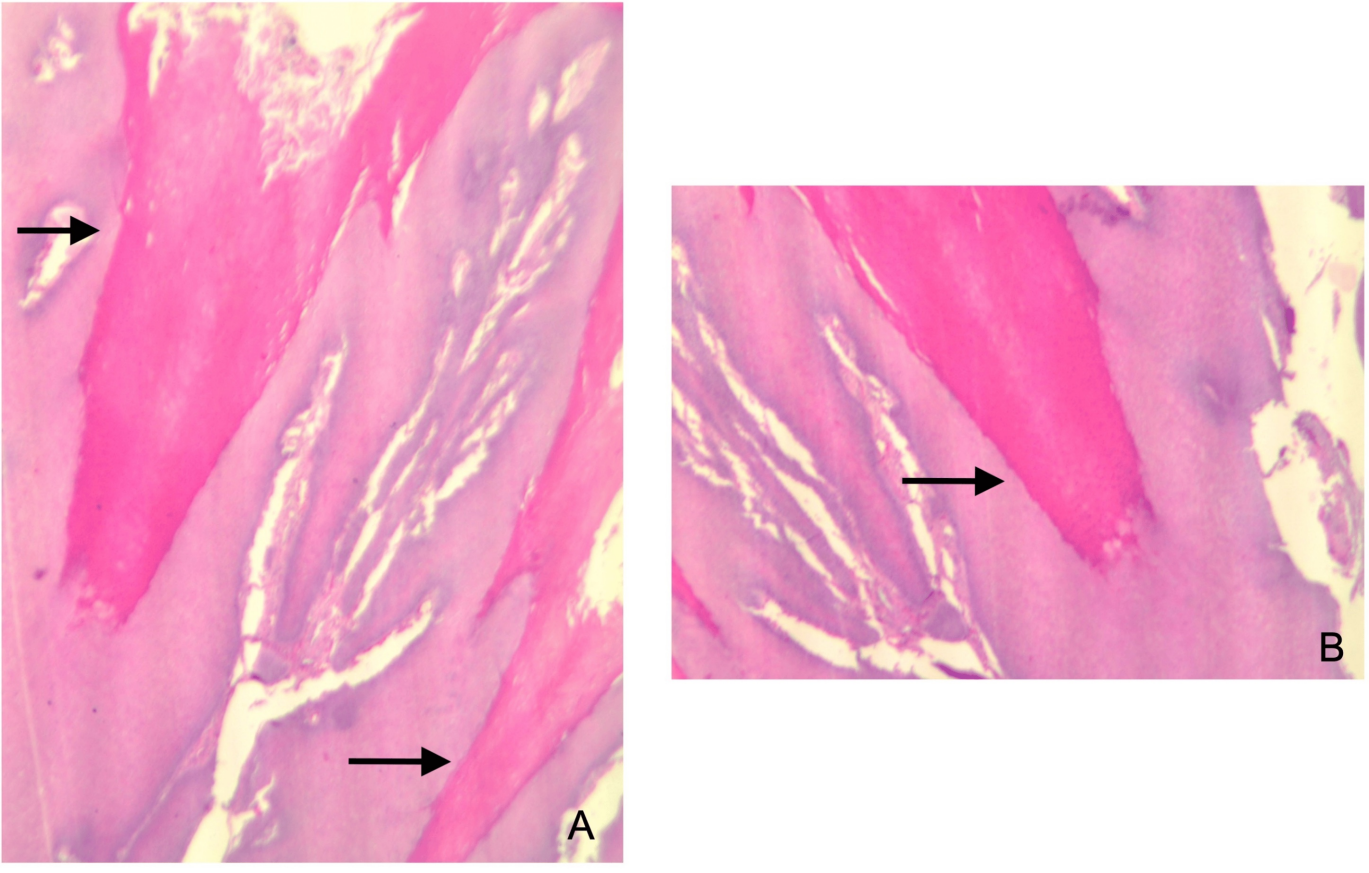
Photomicrographs: parakeratin plugging and bulbous, inferiorly pushing rete pegs with no evidence of dysplasia (A) H&E, 20× and (B) H&E, 40×.

Microscopic examination of the hematoxylin-eosin-stained sections revealed a severely thickened stratified squamous epithelium with a parakeratin layer exhibiting extensive papillary architecture (Figures 2A, 2B). Multiple areas of parakeratin plugging were noted throughout the epithelial proliferation, with pushing margins into the underlying lamina propria. There was no evidence of atypia or dysplasia (Figures 3A, 3B). The rete ridges appeared bulky, broad-based, and acanthotic. These microscopic findings rendered a diagnosis of OVC. Our patient is presently doing well, with no evidence of recurrence seven years after diagnosis. She is on long-term follow-up twice a year.

## Discussion

VC is a slow-growing, asymptomatic, low-grade epithelial malignancy that commonly affects regions of the head and neck, such as the oral cavity, the larynx, and the esophagus [2]. However, it can also appear on the genitalia and the skin [2]. Within the oral cavity, OVC most commonly manifests on the buccal mucosa, followed by the gingiva and the tongue [5]. Approximately 28.9% of OVCs are seen on the tongue [6]. OVC usually occurs in men above the age of 55 years. Among oral malignancies, OVC has a prevalence of 2%-12% [7]. Given its limited metastasis and high level of differentiation, it carries an excellent prognosis [8]. Although the etiology of OVC is complex and its pathogenesis is multifactorial, OVC is more frequently seen in individuals who are chronic snuff users [2]. Additionally, it often develops in patients with a history of tobacco smoking and PVL, a pre-malignant lesion [3]. HPV-6, 11, 16, and 18 have also been detected in some cases of OVC; however, this relationship is not well-established [2]. Because our patient lacked the conventional etiologies, we recommended p16, a marker for high-risk HPV infection. Our patient refused the testing for financial reasons.

Clinically, OVC manifests as a thick, white to erythematous lesion depending on its level of keratinization with an exophytic growth and verrucopapillary projections. The lesion begins as a small papillary area; however, it can progressively extend deeper into local tissues over time [9]. Histologically, it exhibits well-defined, long, and bulbous rete ridges that extend into the underlying connective tissue [2]. The epithelium displays substantial keratin production and does not typically exhibit signs of atypia or dysplasia [2,4]. The appropriate treatment for OVC is surgical excision, although radiotherapy, chemotherapy, or a combination can also be considered [2,7,8]. The success of the surgery depends on the size, location, and duration of the lesion [7]. Due to the clinical resemblance of OVC to other papillary entities, this lesion can be misdiagnosed, possibly delaying optimal treatment. The differential diagnoses for our case included squamous papilloma, condyloma acuminatum, verruciform xanthoma, and verruca vulgaris.

Squamous papilloma

Squamous papilloma is a benign proliferation of the squamous epithelium, commonly induced by HPV-6 and 11 [2]. Intraorally, the lesion may arise anywhere but is frequently seen on the labial mucosa, the tongue, and the palate [10]. It appears clinically as an asymptomatic solitary lesion, enlarging to an average size of 0.5 cm, with white to pink exophytic surface projections [2,8]. In contrast, the lesion that our patient presented with was much larger than 1 cm in size and showed extension beyond the initial area of involvement. Histologically, squamous papilloma exhibits a proliferation of keratinized stratified squamous epithelium with finger-like projections and fibrovascular connective tissue cores [2,8]. The treatment of choice for squamous papilloma is surgical excision [2,8].

Condyloma acuminatum

Condyloma acuminatum is an HPV-induced benign proliferation of the stratified squamous epithelium, affecting the genitals as well as the oral cavity [2]. This entity is most commonly caused by HPV-6, 11, 16, and 18 and is commonly seen in patients with a history of a sexually transmitted disease, including but not limited to HIV, syphilis, and chlamydia. This is in contrast to our patient’s medical history since she denied any history of sexually transmitted diseases. It has a similar papillary appearance to OVC, presenting as an exophytic mass with blunted surface projections, although condyloma acuminatum usually occurs as multiple lesions and forms clusters, which was not evident in our case. The color of the lesion is generally white or lighter than the surrounding tissue [8]. Histologically, it appears as a parakeratotic epithelium with papillary surface projections and numerous koilocytes [2,11]. Condyloma acuminatum can be treated by surgical excision, electrocautery, cryosurgery, photodynamic therapy, and several other methods [11].

Verruciform xanthoma

Verruciform xanthoma is an idiopathic benign proliferation of the epithelium [2]. Although the exact etiology is unknown, it may be caused by trauma or because of other immune-mediated conditions, such as lichen planus, lupus erythematosus, and pemphigus vulgaris [2]. Intraoral verruciform xanthoma predominantly occurs on the gingiva. However, it can be seen extraorally as well, mostly appearing on the genitals [2]. Clinically, it presents as a well-demarcated, papillary, and slightly elevated lesion [2]. Verruciform xanthoma can appear as white, yellow, or red, depending on the amount of keratinization [8]. Although similar to OVC, it rarely grows larger than 2 cm [2]. Microscopically, this lesion is differentiated from OVC by the presence of xanthoma cells; foam cell macrophages found in the papillae of connective tissue [2]. The appropriate treatment is surgical excision [2,8].

Verruca vulgaris

Verruca vulgaris is a focal hyperplasia of squamous epithelium, caused by HPV-2, 4, and 40 [2]. This entity is most often seen on the skin and less commonly in the oral cavity, generally spreading by autoinoculation [2]. It frequently occurs on the skin of the hands and feet, while intraorally it often arises on the vermillion border, anterior tongue, or the labial mucosa [2,10]. Though usually seen in children, it can occasionally appear in older individuals [2]. Clinically, it presents as a painless papule with papillary projections or a rough surface, varying in color between pink, yellow, or white [2]. However, verruca vulgaris does not typically enlarge to a size greater than 0.5 cm [2]. Histologically, it exhibits hyperkeratotic stratified squamous epithelium with elongated rete ridges, focally localized towards the center of the lesion [2]. Verruca vulgaris is treated with liquid nitrogen cryotherapy, topical applications containing salicylic acid and lactic acid, surgical excision, or can undergo spontaneous resolution [2].

**Table 1:** Clinical differential diagnoses of verrucopapillary lesions. PVL, proliferative verrucous leukoplakia; HPV, human papillomavirus

	Verrucous carcinoma	Squamous papilloma	Condyloma acuminatum	Verruciform xanthoma	Verruca vulgaris
Etiology	Tobacco, snuff, PVL, HPV	HPV-6, 11	HPV-6, 11, 16, 18	Trauma, immune-mediated conditions	HPV-2, 4, 40
Age/Sex	>55 years, male predilection	30-50 years, no gender predilection	Sexually transmitted disease, no gender predilection	40-70 years, male predilection	Younger patients, no gender predilection
Clinical features	White, erythematous, pink, papillary, larger, extensive	White, papillary, solitary, small (usually < 0.5 cm)	White, papillary, larger, multiple, clustered	White/yellow, small (maximum = 2 cm)	Pink/yellow/white, papillary, singular, small (< 1 cm)

## Conclusions

Because verrucous carcinoma closely resembles other papillary conditions clinically, general dentists and specialists need to be aware of its clinical features. Misdiagnosed or longstanding cases of VC often display more pronounced changes, such as color variation ranging from white, representing leukoplakia, to red and white, representing erythroleukoplakia, and red, representing erythroplakia. Advanced cases of VC can present as large, thick, and extensively papillary lesions at the time of diagnosis. Therefore, timely diagnosis and complete surgical excision are recommended to prevent recurrence and the development of conventional SCC. This report emphasizes the importance of differentiating VC from other papillary lesions with a cauliflower-like appearance, since VC is a malignancy and potentially exhibits a completely different clinical outcome than the other lesions described here.
